# Glutamate transporter SLC1A1 is associated with clear cell renal cell carcinoma

**DOI:** 10.3906/sag-1808-130

**Published:** 2019-04-18

**Authors:** Sercan ERGÜN, Sezgin GÜNEŞ, Recep BÜYÜKALPELLİ, Oğuz AYDIN

**Affiliations:** 1 Ulubey Vocational Higher School, Ordu University, Ordu Turkey; 2 Department of Medical Biology, Faculty of Medicine, Ondokuz Mayıs University, Samsun Turkey; 3 Department of Urology, Faculty of Medicine, Ondokuz Mayıs University, Samsun Turkey; 4 Department of Pathology, Faculty of Medicine, Ondokuz Mayıs University, Samsun Turkey

**Keywords:** Glutamate transporter, *SLC1A1*, oncogene, renal cell carcinoma

## Abstract

**Background/aim:**

This study aimed to comparatively analyze the expression levels of the *SLC1A1* gene in renal specimens from tumors and adjacent healthy kidney tissues of patients with clear cell renal cell carcinoma (ccRCC).

**Material and methods:**

Nineteen patients diagnosed with ccRCC were included in the study. The expression levels of the *SLC1A1* and *GAPDH* genes were measured in tumor and formalin-fixed paraffin-embedded (FFPE) tissue specimens from the adjacent healthy kidney of each subject. Via the GEPIA database, the distribution of *SLC1A1* gene expressions in ccRCC and healthy kidney tissues was obtained. The relative expression of *SLC1A1* was evaluated for the association with the clinical parameters of the patients.

**Results:**

The expression of the *SLC1A1* gene was significantly higher in males than females (P = 0.029). Also, there were statistically significant associations between stages II–IV and Fuhrman grades 2–4 with respect to *SLC1A1* gene expression (P < 0.001 for both). Moreover, low levels of red blood cell and hemoglobin counts were significantly associated with the *SLC1A1* expression (P < 0.001 and P = 0.005, respectively). The expression of the *SLC1A1* gene in tumor tissues increased approximately 3 times compared with normal kidney tissues (P < 0.05). According to the GEPIA database, *SLC1A1* gene expression is significantly higher in ccRCC patients than healthy persons (P = 0.01).

**Conclusion:**

The change in the expression of *SLC1A1* may be crucial for ccRCC pathophysiology.****

## 1. Introduction

Renal cell carcinoma (RCC) is different from kidney cancer with the involvement of the renal pelvis or renal medullary and it is also the only type of cancer that occurs in cells (renal tubules) that extend into the kidney bed. RCC includes a range of heterogeneous cancers arising from renal tubular cells. RCC is the third most common cause of death after prostate and bladder cancer among the urological cancers and accounts for about 2% of all adult cancer patients. Moreover, its clinical course is the most fatal one among urological cancers. RCC is caused by the accumulation of numerous genetic and epigenetic alterations similar to other cancer types (1).

Clear cell renal cell carcinomas (ccRCCs) are ordinarily globular masses that may originate anywhere in the renal cortex and frequently extrude beyond the normal form of the kidney. Often, ccRCC attacks the renal venous system, sometimes filling the renal vein and growing longer into the vena cava or even the right atrium. It is by far the most frequent type of kidney cancer in adults (2).

In the last twenty years, genetic and clinical studies have presented that ccRCC is both heterogeneous in its histology and clinical course and heterogeneous in its genetic changes (3). The identification of various histological subtypes of ccRCC ensures a better comprehension of the molecular mechanism of these distinct subtypes of cancer and one or more crucial mutations have been defined for each subtype (4). Sporadic cancers originate from multiple (epi)genetic alterations. Therefore, promoter hypermethylation of genes is considered to be involved in sporadic or hereditary forms of ccRCC. The epigenetic alterations that regulate the formation and progression of ccRCC have not been fully revealed yet and are still in their early stages of investigation (5). A more detailed specification of epigenetically changed genes and pathways in ccRCC may lead to the development of new and minimally invasive diagnostic and prognostic tools for ccRCC. For the future, epigenetic therapies may provide an additional treatment preference for advanced ccRCC that does not respond to standard therapy (5). Factors that are potentially related to the pathophysiology of ccRCC and probably usable as biomarkers in the development of the disease need to be investigated.

The *SLC1A1* gene encodes a member of the high-affinity glutamate transporters that function to transport glutamate across plasma membranes. A negatively charged nonessential amino acid, glutamate has been broadly investigated for its function in the central nervous system as a neurotransmitter. Recently, an arising function for metabolic flux and glutamate signaling in cancer has been put forward by in vitro studies. The inhibition of extracellular glutamate binding to ionotropic glutamate receptors (GluRs) impedes the development of many types of cancer, including breast cancer, via apoptosis activation, cell division inhibition, and cell motility reduction. Moreover, intracellular glutamate can be utilized to generate ATP and macromolecules in order to assist cancer cell proliferation and it is necessary for glutathione (GSH) synthesis. Comprehensive studies have focused on glutamine uptake and catabolism (through glutaminase) as the primary source of intracellular glutamate in cancer cells. The metabolic flux of glutamine to glutamate is mediated by oncogenes like RAS and MYC. In spite of the interest in glutamine-glutamate flux as a regulator of the metabolic switch in cancer, limited data exist on the expression and role of glutamate transporters in ccRCC (6).

This study aimed to comparatively analyze the expression levels of the *SLC1A1* gene in renal specimens from tumors and the adjacent healthy kidney tissues of patients with ccRCC and to correlate them with clinical data of patients. 

## 2. Materials and methods

### 2.1. Patient selection 

The study included 19 patients who applied to the Urology Clinic of the Faculty of Medicine at Ondokuz Mayıs University (OMU) between June 2016 and June 2017 for the first time. The inclusion criteria for patient selection were diagnosis with ccRCC and tumor resection with radical nephrectomy. The exclusion criteria included the status of having a tumor measuring 5 mm or less (papillary adenoma); having chronic leukemia or lymphoma, inflammatory disease, or autoimmune disease; receiving neoadjuvant or adjuvant therapy; having other coexisting subtypes of RCC; and a previous history of RCC. The number of patients to be included in the study was detected with a test power of 80% and a confidence interval of 95%. The statistically significant number of patients was calculated as at least 19.

Histopathological examination was performed on the tumor tissue and some of the healthy kidney tissues around this tumor resected by radical nephrectomy. Formalin-fixed paraffin-embedded (FFPE) tissues were stored as archival tissues and kept at room temperature at the Department of Pathology. The results of histopathological and radiological examinations of the patients were used for the diagnosis of ccRCC. The 2012 Vancouver Renal Neoplasia Classification of the International Society of Urological Pathology was used for the histological classification of renal tumors of the patients. 

The Clinical Investigation Ethics Committee of OMU approved the study and written informed consent was received from the subjects participating in the study (Approval No: 2016/139).

### 2.2. RNA isolation from FFPE tissues

Excess paraffin was removed from over the tumor and the adjacent healthy kidney FFPE tissue specimens of each subject in the archive. Then 3 or 4 sections of 5 µm in thickness were taken using a microtome (Leica Microsystems, Nussloch, Germany) and transferred to sterile 1.5-mL microcentrifuge tubes. The miRNeasy FFPE Kit (QIAGEN GmbH, Hilden, Germany) was used for the total RNA isolation from FFPE tissue sections. 

### 2.3. cDNA synthesis and measurement of cDNA concentration

The obtained RNA samples were converted to cDNA by reverse transcription using the Ipsogen RT Kit (QIAGEN). The cDNA samples were stored at –20 °C until real-time polymerase chain reaction (RT-PCR) was performed. 

The spectrophotometric method was used to determine the quality and concentration of the cDNA samples obtained before RT-PCR experiments. This analysis was performed using a microplate spectrophotometer (Multiscan GO, Thermo Scientific, USA). 

### 2.4. Gene expression analysis

The RT-PCR method was used for gene expression analysis and Rotor Gene Q (QIAGEN) was used for this purpose.

A primer pair specific to the *SLC1A1* gene was used in the expression analysis, which was the Hs_SLC1A1_1_SG QuantiTect Primer Assay (QIAGEN). As an internal control, the housekeeping *GAPDH* gene and the primer pair specific to it (Hs_GAPDH_1_SG QuantiTect Primer Assay) (QIAGEN) was used. RT2 SYBR® Green qPCR Mastermix (QIAGEN) was used as premix for the gene expression analysis. 

### 2.5. GEPIA database analysis

The distribution of *SLC1A1* gene expressions in ccRCC and healthy kidney tissues according to TCGA normal and GTEx data was schematized by the GEPIA database (7). Also, the list of most differential survival genes in ccRCC was generated by using the GEPIA database in order to see the order of the *SLC1A1* gene with respect to the differential survival association in ccRCC.

### 2.6. Statistical analysis

Tumor samples from patients with RCC were compared with the healthy tissue in the periphery of the tumor tissue of the same patient. Using the Ct values obtained from these tissues, the expression levels of the respective genes were compared statistically. In the method based on partial quantities, the measured values of the expression measurement genes were normalized by *GAPDH* transcription. For the comparison, Ct values were obtained and the 2–ΔΔCt formula were used: 

2–ΔΔCt = 2–[Tumor ΔCt (Gene – Reference) – Control ΔCt (Gene – Reference)] (1)

The 2–ΔCt formula was used to calculate gene expression levels separately for the tumor and the adjacent healthy kidney tissue. Using this formula, the fold changes in the expression of genes in the tumor and the adjacent healthy kidney tissue relative to the reference gene were calculated separately. Statistical analysis of the significance of fold changes in gene expression levels obtained from this formula was then performed.

2–ΔCt = 2–[Tumor ΔCt (Gene – Reference)] (2)

2–ΔCt = 2–[Control ΔCt (Gene – Reference)] (3)

All statistical analyses were performed using SPSS 21 (IBM Corp., Armonk, NY, USA). Normal distribution of data was evaluated statistically by the Kolmogorov–Smirnov test. It was decided to use nonparametric tests because the data were not suitable for normal distribution (P < 0.05) and the number of samples was below 30. The Wilcoxon signed-rank test was used in binary comparisons and the Kruskal–Wallis test was applied in multiple comparisons. P < 0.05 was accepted as a statistically significant value and the evaluation was made at 0.95 confidence interval.

## 3. Results

The expression levels of the *SLC1A1* gene in tumor tissues and the healthy kidney tissues surrounding the tumors of 19 ccRCC patients were compared and possible associations between the clinical parameters of the patients and gene expression levels were investigated.

Demographic (sex, age) and clinicopathological (TNM staging, Fuhrman nuclear grade, red blood cell (RBC) count, hemoglobin count) characteristics of the patients enrolled in this study are presented in Table 1. The expression of the *SLC1A1* gene was significantly higher in males than females (P = 0.029). Also, there were statistically significant associations between stages II–IV and Fuhrman grades 2–4 with respect to *SLC1A1* gene expressions (P < 0.001 for both). Moreover, low levels of RBC and hemoglobin counts, which were parameters giving information about the occurrence of ccRCC, are significantly associated with *SLC1A1* expression (P < 0.001 and P = 0.005, respectively).

**Table 1:** Demographic and clinicopathological characteristics of patients and the statistical significance of the associations of these data with SLC1A1 gene expressions of the patients

		Patients (n = 19)	P-value
Sex	Male	9 (47.4%)	0.029
	Female	10 (52.6%)	
Age	35–44	4 (21%)	0.584
	45–64	10 (52.7%)	
	>65	5 (26.3%)	
TNM staging	Stage I	13 (68.4%)	<0.001 (between stage II and IV)
	Stage II	3 (15.8%)	
	Stage III	1 (5.3%)	
	Stage IV	2 (10.5%)	
Fuhrman nuclear grade
	Grade 2	13 (68.4%)	
	Grade 3	2 (10.5%)	<0.001 (between grade 2 and 4)
Grade 4	4 (21.1%)	
RBC count	Low	4 (21%)	<0.001 (between low and normalRBC count)
	Normal	14 (73.7%)	
	High	1 (5.3%)	
Hemoglobin count	Low	7 (36.8%)	0.005 (between low and normal hemoglobin count)
	Normal	12 (63.2%)	

TNM: Tumor-Node-Metastasis. RBC count lower than 4 million/mm3 and hemoglobin count lower than 13 g/dL were considered as low.

**Figure 1:**
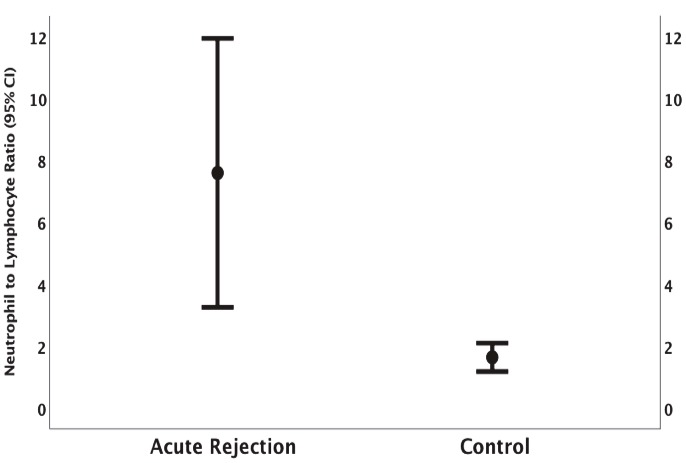
Quantitative expression levels of the SLC1A1 gene in tumor tissues as compared with adjacent healthy kidney tissues of patients with ccRCC (P < 0.05).

**Figure 2:**
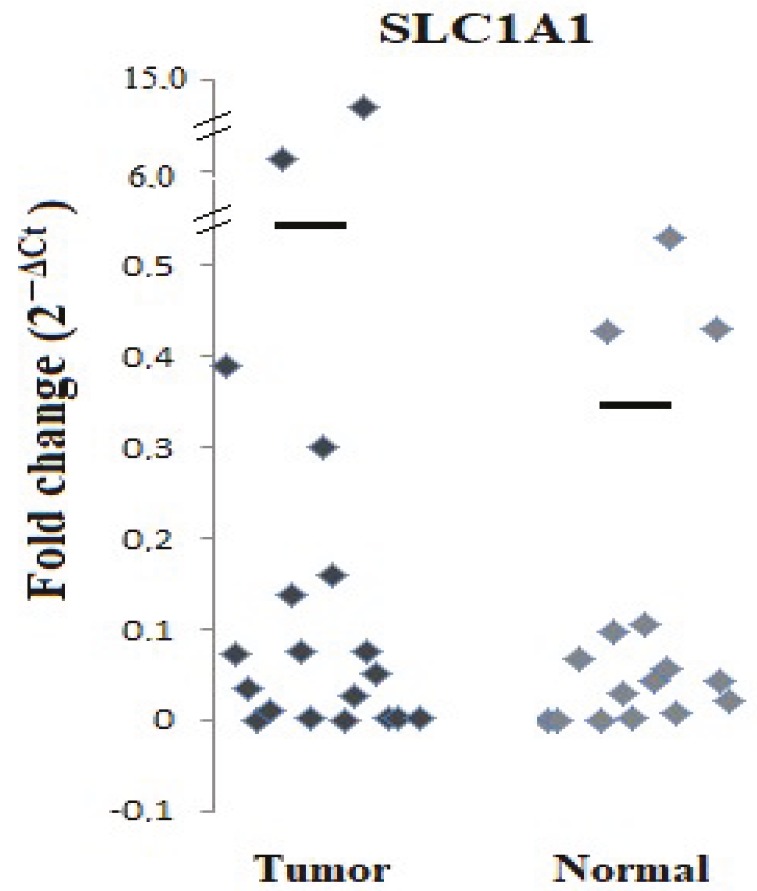
Distribution of patients’ quantitative expression levels of the SLC1A1 gene in tumor tissues as compared to adjacent healthy kidney tissues.

Tumor samples of patients with ccRCC were compared with healthy kidney tissues around the tumor of the same patient with respect to the expression levels of the *SLC1A1 *gene (Figures 1 and 2). The expression of the *SLC1A1* gene in kidney tumor tissues increased approximately 3 times compared with normal kidney tissues. As shown in Figures 1 and 2, the change in the expression levels of the *SLC1A1 *gene between the tumor and normal kidney tissues was statistically significant (P < 0.05). 

**Figure 3:**
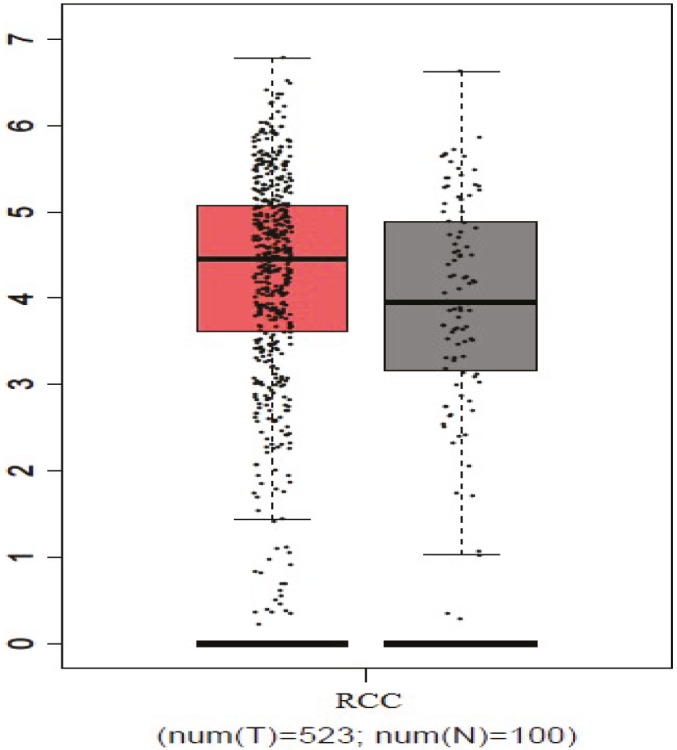
Distribution of SLC1A1 gene expressions in ccRCC and healthy kidney tissues according to TCGA normal and GTEx data in GEPIA database (P = 0.01).

According to the GEPIA database, which is based on TCGA normal and GTEx data of 523 ccRCC patients and 100 healthy persons, *SLC1A1* gene expression is significantly higher in ccRCC patients when compared with healthy persons (P = 0.01) (Figure 3). As can be seen, both the present study and the GEPIA database show significantly increased levels of *SLC1A1* gene expression in ccRCC cases when compared with healthy control cases. In other words, the outcomes of the GEPIA database support our findings. 

Lastly, a list of genes was extracted as the most differential survival genes in ccRCC according to the GEPIA database (Table 2). *SLC1A1* was one of these genes. This shows a significant relationship of *SLC1A1* and supports our findings. 

**Table 2:** The list of most differential survival genes in ccRCC according to GEPIA database.

Gene symbol	Gene ID	P-value (overall survival)
PEBP1P2	ENSG00000270532.1	1.85e-14
RP11-379F4.8	ENSG00000271778.1	3.42e-14
SOWAHB	ENSG00000186212.3	1.84e-13
CPT2	ENSG00000157184.5	1.92e-13
WDR72	ENSG00000166415.14	2.25e-13
DHRS12	ENSG00000102796.10	1.30e-12
TOLLIP	ENSG00000078902.15	1.44e-12
TPRG1L	ENSG00000158109.14	1.59e-12
BAG1	ENSG00000107262.16	3.58e-12
SLC1A1	ENSG00000106688.11	5.68e-12

## 4. Discussion

Renal cell carcinoma is one of the 15 most frequent types of cancer arising globally. The most aggressive subtype, ccRCC comprises about 70% of all kidney tumors. ccRCC is potentially treatable by resection. However, approximately 30% of patients show recurrence after the first nephrectomy. Unfortunately, ccRCC is often nonsymptomatic in the early stages and is frequently reported in the advanced phase with metastases. In the case of metastasis, ccRCC is radiation- and chemoresistant and remains incurable in most cases, resulting in a mortality rate of 95%. To date, no effective ccRCC therapy has been created and none of the probable biomarkers have been approved for clinical administration (8). 

In this study, tumor tissues of the patients with ccRCC were compared with healthy kidney tissues around the tumor tissues of the same patients in terms of the expression levels of the *SLC1A1* gene and possible associations between the clinical parameters of the patients and gene expression levels were analyzed.

According to the association analysis between demographic (sex, age) and clinicopathological (TNM staging, Fuhrman nuclear grade, RBC count, hemoglobin count) parameters and the expression level of the *SLC1A1* gene, interesting results were obtained. The expression of the *SLC1A1* gene was significantly higher in males than in females (P = 0.029). Also, there were statistically significant associations between stages II–IV and Fuhrman grades 2–4 with respect to *SLC1A1* gene expression (P < 0.001 for both). Moreover, low levels of RBC and hemoglobin counts, which were parameters giving information about the occurrence of ccRCC, are significantly associated with *SLC1A1* expression (P < 0.001 and P = 0.005, respectively). Most studies in the literature support our findings with respect to the relationship of *SLC1A1* expression in ccRCC with anemia. Anemia is a condition in which individuals have insufficient healthy RBCs to carry adequate oxygen to tissues in the body. According to a study that retrospectively reviewed 204 patients diagnosed with ccRCC between 1995 and 2008 in a community hospital setting, anemia can precede the diagnosis of ccRCC. Upon the exclusion of the measurement of hemoglobin levels from the study, the severity of anemia corresponds to poorer overall survival of ccRCC patients (9). On the contrary, it is known that kidney cancer cells can release a hormone called erythropoietin. This hormone causes the bone marrow to produce too many RBCs. In other tissues, especially in cancer cells, it inhibits apoptosis, stimulates angiogenesis, and promotes cell proliferation. Many studies have confirmed the overexpression of erythropoietin and its receptor in clear cell renal carcinoma (10). 

Tumor samples of patients with ccRCC were compared with healthy kidney tissues around the tumor of the same patient with respect to the expression level of the *SLC1A1 *gene. The expression of the *SLC1A1* gene in kidney tumor tissues increased approximately 3 times compared with normal kidney tissues, which was statistically significant. Also, according to the GEPIA database, which is based on TCGA normal and GTEx data of 523 ccRCC patients and 100 healthy persons, *SLC1A1* gene expression is significantly higher in ccRCC patients when compared with healthy persons in correlation with our wet-lab experiments. The studies in the literature seem to support our results. A study investigating signaling through glutamate receptors in human cancers showed that increased activity of hypoxia-inducible factors (HIFs) due to hypoxia or von Hippel–Lindau (VHL) loss-of-function in ccRCC augmented the release of glutamate, which was mediated by HIF-dependent expression of the *SLC1A1* and *SLC1A3* genes encoding glutamate transporters (11). When looking at the association between *SLC1A1* and cancer types other than ccRCC, a significant correlation was observed between *SLC1A1* overexpression and glioma. According to this association, the uptake of neurotransmitter glutamate is affected primarily by transporters expressed on astrocytes, and the downregulation of these transporters leads to seizures and neuronal death. Neurons also express a glutamate transporter, excitatory amino acid carrier-1 (*SLC1A1*), but the physiological function of this transporter remains uncertain. In a study conducted on C6 rat glioma cells, a line described to contain neural stem-like cells, *SLC1A1* was markedly induced by all-*trans* retinoic acid (ATRA), a well-known differentiating agent. After 4 days of treatment with 10 µM ATRA, *SLC1A1* mRNA was increased by 400% compared with the control, and the C6 culture was greatly enriched of cells with bipolar morphology strongly positive for *SLC1A1* immunoreactivity. Thus, the increased expression of *SLC1A1* was correlated with the differentiation of glioma (12). In another study, genetically *SLC1A1*-null (Slc1a1−/−) mice had reduced levels of neuronal glutathione and, with aging, developed brain atrophy and behavioral changes. Therefore, *SLC1A1*-knockout mice exhibit increased oxidative stress due to GSH depletion (13). Also, a study conducted with 68 patients with renal tumors and 30 age-matched normal controls detected significantly increased reactive oxygen species and nitric oxide and decreased glutathione in patients with renal cell carcinoma compared with normal subjects (14). When we evaluate these two studies together, it is possible to infer that ccRCC causes oxidative stress and shows this effect via increased levels of *SLC1A1* expression. 

A list of genes was extracted as the most differential survival genes in ccRCC according to the GEPIA database and *SLC1A1* was seen as one of these genes. This also supports significant associations between *SLC1A1* and ccRCC, as in our results. 

In conclusion, the changes in the expression levels of the genes analyzed and RCC-specific parameters need to be confirmed in larger study groups. If verified, it may be possible to use the expression changes of *SLC1A1* as biomarkers for the prognosis of ccRCC. The results of this study may suggest that molecular applications can be designed to change the level of the expression of *SLC1A1* to affect the development of ccRCC in future projects.****

As the limitations of the study, perhaps the greatest source of complexity and variability in gene expression experiments stems from phenomena not related to treatment, or intrinsic variability, which is hard to control and reproduce. Normal fluctuations in gene expression will occur as a result of differences in age, sex, temperature, light, diet, and hormonal status. Although age, sex, and the external environment can be tightly controlled within experiments, comparisons between laboratories using similar protocols may be more challenging when environmental factors are not strictly controlled. Differences in nutritional or hydration status, time of last meal, hormonal fluctuations during estrus, and seasonal and light-induced changes in hormone levels are more difficult to control within experiments.
